# Valid publication of names for bacterial species from the chicken gut

**DOI:** 10.1099/ijsem.0.006445

**Published:** 2024-07-05

**Authors:** Mark J. Pallen

**Affiliations:** 1 Quadram Institute Bioscience, Norwich Research Park, Norwich, UK

**Keywords:** chicken gut microbiome, prokaryotic taxonomy, prokaryotic nomenclature

## Abstract

This article is a follow-up to Gilroy R, Ravi A, Getino M, Pursley I, Horton DL, *et al*. *PeerJ* 2021;9:e10941, detailing accession numbers from culture collections to ensure that names for 33 new species conform to the Rules of the International Code of Nomenclature of Prokaryotes required for valid publication of names for cultured species. The following species names are now proposed to be recognized as validly published: *Acinetobacter pecorum* sp. nov., *Arthrobacter gallicola* sp. nov., *Arthrobacter pullicola* sp. nov., *Bacillus norwichensis* sp. nov., *Brevibacterium gallinarum* sp. nov., *Brevundimonas guildfordensis* sp. nov., *Cellulomonas avistercoris* sp. nov., *Clostridium gallinarum* sp. nov., *Comamonas avium* sp. nov., *Corynebacterium gallinarum* sp. nov., *Cytobacillus stercorigallinarum* sp. nov., *Escherichia whittamii* sp. nov., *Kaistella pullorum* sp. nov., *Luteimonas colneyensis* sp. nov., *Microbacterium commune* sp. nov., *Microbacterium gallinarum* sp. nov., *Microbacterium pullorum* sp. nov., *Oceanitalea stevensii* sp. nov., *Ochrobactrum gallinarum* sp. nov., *Oerskovia douganii* sp. nov., *Oerskovia gallyi* sp. nov., *Oerskovia merdavium* sp. nov., *Oerskovia rustica* sp. nov., *Paenibacillus gallinarum* sp. nov., *Phocaeicola gallinarum* sp. nov., *Planococcus wigleyi* sp. nov., *Psychrobacter communis* sp. nov., *Serpens gallinarum* sp. nov., *Solibacillus faecavium* sp. nov., *Sporosarcina gallistercoris* sp. nov., *Sporosarcina quadrami* sp. nov., *Stenotrophomonas pennii* sp. nov. and *Ureibacillus galli* sp. nov.

## Valid publication of names for new bacterial species from the chicken gut

Protologues for the following species have been published in Gilroy *et al*. [1]. However, at that time, there were no accession numbers available from culture collections to allow valid publication of names. The following table now provides sufficient information for valid publication of the relevant names.

**Table:** 

Name	Described as	Etymology	Properties	Nomenclatural type	GenBank ID of type strain genome	Deposited as
*Acinetobacter pecorum*	sp. nov.	pe.co'rum. L. gen. pl. n. *pecorum,* of flocks of sheep, birds etc., as this species has been isolated from chickens and sheep	As described in Gilroy *et al*. [1]	Sa1BUA6	GCA_014837015.1.	DSM 112314, NCTC 14545
*Arthrobacter gallicola*	sp. nov.	gal.li'co.la. L. masc. n. *gallus,* a cock; N.L. suff. *-cola* an inhabitant of; N.L. masc. or fem. n. *gallicola*, an inhabitant of the chicken	As described in Gilroy *et al*. [1]	Sa2CUA1	GCA_014836775.1.	DSM 112320, NCTC 14560
*Arthrobacter pullicola*	sp. nov.	pul.li'co.la. L. masc. n. *pullus*, a young chicken; N.L. suff. *-cola* an inhabitant of; N.L. masc. or fem. n. *pullicola*, an inhabitant of young chickens	As described in Gilroy *et al*. [1]	Sa2BUA2	GCA_014836875.1.	DSM 112319, NCTC 14559
*Bacillus norwichensis*	sp. nov.	nor.wich.en’sis. N.L. masc. adj. *norwichensis*, pertaining to English city of Norwich, where the organism was isolated	As described in Gilroy *et al*. [1]	Sa1BUA2	GCA_014836955.1.	DSM 112315, NCTC 14546
*Brevibacterium gallinarum*	sp. nov.	gal.li.na'rum. L. gen. pl. n. *gallinarum*, of hens	As described in Gilroy *et al*. [1]	Re57	GCA_014836885.1.	DSM 112317, NCTC 14547
*Brevundimonas guildfordensis*	sp. nov.	guild.ford.en’sis. N.L. fem. adj. *guildfordensis,* pertaining to English town Guildford, home to the University of Surrey, where the samples were processed	As described in Gilroy *et al*. [1]	Sa3CVA3	GCA_014836405.1.	DSM 112318, NCTC 14558
*Cellulomonas avistercoris*	sp. nov.	a.vi.ster'co.ris. L. fem. n. *avis*, bird; L. neut. n. *stercus* dung; N.L. gen. n. *avistercoris*, of bird faeces	As described in Gilroy *et al*. [1]	Sa3CUA2	GCA_014836445.1.	DSM 112321, NCTC 14564
*Clostridium gallinarum*	sp. nov.	gal.li.na'rum. L. pl. gen. n. *gallinarum*, of hens	As described in Gilroy *et al*. [1]	Sa3CUN1	GCA_014836325.1.	DSM 112313, NCTC 14701
*Comamonas avium*	sp. nov.	a'vi.um. L. gen. pl. n. *avium*, of birds	As described in Gilroy *et al*. [1]	Sa2CVA6	GCA_014836675.1.	NCTC 14571, CCUG 77452
*Corynebacterium gallinarum*	sp. nov.	gal.li.na'rum. L. pl. gen. n. *gallinarum*, of hens	As described in Gilroy *et al*. [1]	Sa1YVA5	GCA_014837045.1.	DSM 112355, NCTC 14569
*Cytobacillus stercorigallinarum*	sp. nov.	ster.co.ri.gal.li.na'rum. L. neut. n. *stercus,* faeces; L. fem. n. *gallina*, a hen; N.L. gen. n. *stercorigallinarum,* of hen faeces	As described in Gilroy *et al*. [1]	Sa5YUA1	GCA_014836495.1.	DSM 112322, NCTC 14702
*Escherichia whittamii*	sp. nov.	whit.tam'i.i. N.L. gen. n. *whittamii*, named in honour of American microbiologist Thomas S. Whittam	As described in Gilroy *et al*. [1]	Sa2BVA5	GCA_014836715.1.	DSM 112323, NCTC 14567
*Kaistella pullorum*	sp. nov.	pul.lo'rum. L. gen. pl. n. *pullorum* of chickens	As described in Gilroy *et al*. [1]	Sa1CVA4	GCA_014837035.1.	NCTC 14563 CCUG 77451
*Luteimonas colneyensis*	sp. nov.	col.ney.en’sis. N.L. fem. adj. *colneyensis,* pertaining to the English village of Colney, home to the Quadram Institute, where the species was first described	As described in Gilroy *et al*. [1]	Sa2BVA3	GCA_014836665.1.	DSM 112464, NCTC 14570
*Microbacterium commune*	sp. nov.	com.mu'ne. L. neut. adj. *commune,* common, referring to diverse habitats, as this species has been isolated from mosquitos and chicken	As described in Gilroy *et al*. [1]	Re1	GCA_014836945.1.	DSM 112387, NCTC 14561
*Microbacterium gallinarum*	sp. nov.	gal.li.na'rum. L. gen. pl. n. *gallinarum*, of hens	As described in Gilroy *et al*. [1]	Sa1CUA4	GCA_014837165.1.	DSM112389, NCTC 14568
*Microbacterium pullorum*	sp. nov.	pul.lo'rum. L. gen. pl. n. *pullorum*, of chickens	As described in Gilroy *et al*. [1]	Sa4CUA7	GCA_014836535.1.	DSM 112390, NCTC 14562
*Oceanitalea stevensii*	sp. nov.	ste.ven’si.i. N.L. gen. n. *stevensii*, named in honour of British microbiologist Mark Stevens	As described in Gilroy *et al*. [1]	Sa1BUA1	GCA_014837105.1.	DSM 112381, NCTC 14557
*Ochrobactrum gallinarum*	sp. nov.	gal.li.na'rum. L. gen. pl. n. *gallinarum*, of hens	As described in Gilroy *et al*. [1]	Sa2BUA5	GCA_014836735.1.	DSM 112359, NCTC 14555
*Oerskovia douganii*	sp. nov.	dou.gan’i.i. N.L. gen. n. *douganii*, named in honour of British microbiologist Gordon Dougan	As described in Gilroy *et al*. [1]	Sa1BUA8	GCA_015142735.1.	DSM 112378, NCTC 14549
*Oerskovia gallyi*	sp. nov.	gal'ly.i. N.L. gen. n. *gallyi*, named in honour of British microbiologist David Gally	As described in Gilroy *et al*. [1]	Sa2CUA8	GCA_014836745.1.	DSM 112379, NCTC 14550
*Oerskovia merdavium*	sp. nov.	merd.a'vi.um. L. fem. n. *merda*, faeces; L. fem. n. *avis* bird; N.L. gen. n. *merdavium,* of bird faeces	As described in Gilroy *et al*. [1]	Sa2CUA9	GCA_014836755.1.	DSM 112358, NCTC 14554
*Oerskovia rustica*	sp. nov.	rus'ti.ca. L fem. adj. *rustica* of the countryside, as isolates have been obtained from soil and chickens	As described in Gilroy *et al*. [1]	Sa4CUA1	GCA_014836555.1.	DSM 112380, NCTC 14700
*Paenibacillus gallinarum*	sp. nov.	gal.li.na'rum. L. gen. pl. n. *gallinarum*, of hens	As described in Gilroy *et al*. [1]	Sa2BVA9	GCA_014836635.1.	DSM 112363, NCTC 14573, CCUG 77453
*Phocaeicola gallinarum*	sp. nov.	gal.li.na'rum. L. gen. pl. n. *gallinarum*, of hens	As described in Gilroy *et al*. [1]	Sa1YUN3	GCA_014837055.1.	DSM 112465, NCTC 14574
*Planococcus wigleyi*	sp. nov.	wig’ley.i. N.L. masc. gen. n. *wigleyi*, named in honour of British microbiologist Paul Wigley	As described in Gilroy *et al*. [1]	Sa1BUA13	GCA_014836985.1.	DSM 112356, NCTC 14552
*Psychrobacter communis*	sp. nov.	com.mu'nis. L. masc. adj. *communis*, common, referring to diverse habitats from which this species has been isolated, including chickens and soil	As described in Gilroy *et al*. [1]	Sa4CVA2	GCA_014836505.1.	DSM 112357, NCTC 14553
*Serpens gallinarum*	sp. nov.	gal.li.na'rum. L. gen. pl. n. *gallinarum*, of hens	As described in Gilroy *et al*. [1]	Sa2CUA2	GCA_014836765.1.	DSM 112377, NCTC 14618
*Solibacillus faecavium*	sp. nov.	faec.a'vi.um. L. fem. n. *faex*, *faecis*, faeces; L. fem. n. *avis*, bird; N.L. gen. n. *faecavium* of bird faeces	As described in Gilroy *et al*. [1]	A46	GCA_014836905.1.	NCTC 14578, CCUG 77455
*Sporosarcina gallistercoris*	sp. nov.	gal.li.ster'co.ris. L. masc. n. *gallus*, a cock; L. neut. n. *stercus* dung; N.L. gen. n. *gallistercoris,* of faeces of a cock	As described in Gilroy *et al*. [1]	Sa3CUA8	GCA_014836415.1.	DSM 112360, NCTC 14551
*Sporosarcina quadrami*	sp. nov.	qua.dra'mi. N.L. gen. n. *quadrami,* of the Quadram Institute, where the species was first cultured.	As described in Gilroy *et al*. [1]	Sa2YVA2	GCA_014836615.1.	DSM 112361, NCTC 14703
*Stenotrophomonas pennii*	sp. nov.	pen'ni.i. N.L. gen. n. *pennii*, named in honour of British microbiologist Charles W. Penn	As described in Gilroy *et al*. [1]	Sa5BUN4	GCA_014836545.1.	NCTC 14577, CCUG 77454
*Ureibacillus galli*	sp. nov.	gal'li. L. masc. gen. n. *galli*, of a chicken	As described in Gilroy *et al*. [1]	Re31	GCA_014836845.1.	DSM 112365, NCTC 14576
